# Cholera on Blackwell’s Island

**Published:** 1866-10

**Authors:** 


### SELECTED ARTICLES.

*Cholera on Blackwell's Island.*—The following account of the epidemic in this locality, by Prof. Hamilton, will be read with interest.

No. 64 Madison Avenue,
New York, Friday, Aug. 10, 1866.

*E. Harris, M. D., Corresponding Secretary, M. B. H.:*

Sir: The first case of cholera occurred in the Workhouse on the 28th of July; the last case on the 6th of August. The epidemic continued, therefore, nine days, during which period, of about 800 inmates, 123 died. I do not mention one case reported on the 8th of August, because, as I understand, the person was admitted only the night before; I do not think the disease was contracted in the Workhouse.

You know the building very well. It is admirably constructed for the purposes for which it is designed, and, so far as my observation extends, it is always perfectly clean. Until now, the inmates have been as healthy as this class of people are usually found to be.

The explanation of the rapid propagation and fatality of the disease after it had once gained admission, was believed to be confinement and crowding. It was observed that the cholera was for several days exclusively among the women. The women had the smallest apartments, were most crowded in their cells, and, with few exceptions, were employed within the building in close contact with each other during the day. The men were employed mostly in the quarries, out doors.

On Wednesday, when the epidemic was at its height, the first of August, I gave my pledge to the Board of Commissioners and to Mr. Schultz, President of the Board of Health, in your presence, that I would drive the cholera from the Workhouse in from three to five days. I said this in no spirit of boasting, but in simple reliance on the well-known and established laws of Hygiene. The Commissioners executed literally and promptly every order which was given by the Committee.

The epidemic began to decline from the day they were fully carried out, and on Monday last the pledge was redeemed. The following is a summary of the sanitary means adopted:

The inmates were distributed as far as the vacant places in the building would permit; the cell doors were left open at night; the night-buckets were supplied with disinfectants and left outside; the women's cooking rooms were converted into hospital wards, and the women were kept out of doors from morning until night; corn meal and molasses were taken from the diet table; coffee, tea and vegetables were added; at night each inmate was required to take whisky one ounce, water three ounces, tincture of capsicum fifteen drops. [These people are our city vagrants, and probably are habitually intemperate.] A variety of disinfectants were employed freely and constantly in every vessel and closet which received the excreta, even the excreta from the stomach were disinfected immediately after they were received into a vessel or fell upon the floor; stoves were placed in each hospital ward to insure a draft; all windows were kept open night and day; the clothing taken from the cholera patients was sent directly to the boilers; a ward was established for patients with the diarrhœa, and the value of this measure is shown by the fact that of the large number received into this ward only one died. It was difficult, however, to persuade these poor creatures to report themselves at this stage of the disease.

From the Workhouse the cholera has spread to every other building on the Island, except, I think, to the "Madhouse," the pavilion attached to the Male Almshouse and the Fever Pavilion. In none, however, has it proved so fatal as in the Workhouse.

The same sanitary measures have been adopted, with slight modifications, in each department, but they cannot be applied with so much vigor to the Lunatic Asylum, the Almshouse, or the General Hospital. These buildings are all crowded, and the inmates cannot be scattered or turned out of doors; consequently, the cholera remains among them, but in a greatly mitigated form. In the Penitentiary it remained but two days.

Connected with the Almshouse are two well-constructed pavilions, lying side by side, separated only by a few feet and a brick wall 10 or 12 feet high. One is occupied by feeble old men, the other by the same class of old women. The only point of difference which I can discover is, that at the time of the outbreak of the cholera, the male pavilion contained only 62 persons, while the female contained 99. In the first there has not been one case of cholera, in the second 31 have died.

Of 14 house-physicians and surgeons employed in these several buildings, some of whom have been in constant attendance upon the sick, not one has suffered from the epidemic.

Very respectfully yours,

FRANK H. HAMILTON, M. D.

—*N. Y. Med. Rec.*

---

Professor Charles T. Chandler, of the Columbia College School of Mines, is appointed Professor of Chemistry in the New York College of Pharmacy.

